# Encouraging rational antibiotic use in childhood pneumonia: a focus on Vietnam and the Western Pacific Region

**DOI:** 10.1186/s41479-017-0031-4

**Published:** 2017-04-25

**Authors:** Nguyen T. K. Phuong, Tran T. Hoang, Pham H. Van, Lolyta Tu, Stephen M. Graham, Ben J. Marais

**Affiliations:** 1 Respiratory Department, Da Nang Hospital for Women and Children, Da Nang, Vietnam; 2 Neonatal Department, Da Nang Hospital for Women and Children, Da Nang, Vietnam; 3 Microbiology Department, The University of Medicine and Pharmacy, Ho Chi Minh, Vietnam; 4 Antimicrobial Stewardship Team, The Children’s Hospital at Westmead, Sydney, Australia; 5 Centre for International Child Health, University of Melbourne and Murdoch Children’s Research Institute, Melbourne, Australia; 6 Infectious Disease Team, The Children’s Hospital at Westmead and Discipline of Paediatrics and Adolescent Medicine, University of Sydney, Sydney, NSW Australia

## Abstract

Globally, pneumonia is considered to be the biggest killer of infants and young children (aged <5 years) outside the neonatal period, with the greatest disease burden in low- and middle-income countries. Optimal management of childhood pneumonia is challenging in settings where clinicians have limited information regarding the local pathogen and drug resistance profiles. This frequently results in unnecessary and poorly targeted antibiotic use. Restricting antibiotic use is a global priority, particularly in Asia and the Western Pacific Region where excessive use is driving high rates of antimicrobial resistance. The authors conducted a comprehensive literature review to explore the antibiotic resistance profile of bacteria associated with pneumonia in the Western Pacific Region, with a focus on Vietnam. Current management practices were also considered, along with the diagnostic dilemmas faced by doctors and other factors that increase unnecessary antibiotic use. This review offers some suggestions on how these issues may be addressed.

## Background

Childhood pneumonia is a major contributor to under-5 mortality, especially in developing countries [1, 2]. In the Western Pacific Region, the highest burden of childhood pneumonia and pneumonia-related deaths occur in six countries: Cambodia, China, Laos, Papua New Guinea, the Philippines and Vietnam [3]. These countries report at least 0.2 pneumonia episodes per child year—more than 10 times the rates reported in developed countries from the same region such as Australia, New Zealand and Japan [4]. The management of childhood pneumonia is problematic in settings where a microbiological diagnosis is rarely pursued and the drug resistance profiles of common bacteria which cause respiratory disease are not readily available [5]. In many Asian countries this encourages excessive use of broad-spectrum antibiotics, irrespective of the child’s disease severity. In the authors’ experience, children are frequently hospitalized with pneumonia to administer intravenous antibiotics, despite relatively mild disease.

Unnecessary hospitalization for intravenous antibiotics increases healthcare cost, as well as treatment-related complications and the likelihood of nosocomial disease transmission. In order to encourage more rational use of intravenous antibiotics in children with community-acquired pneumonia, the authors performed a comprehensive review of common bacterial pathogens and their reported drug resistance profiles in the Western Pacific Region—Vietnam in particular. Factors that increase unnecessary antibiotic use (and measures that might reduce use) were also considered. PubMed, Google Scholar and Embase databases were searched using the following terms: antibacterial agents OR antibiotics OR drug therapy AND community acquired pneumonia OR acute respiratory tract infection AND child OR children OR childhood. Manuscript titles and abstracts were reviewed to identify original research papers that included children less than 5 years of age with pneumonia, with a geographic focus on Vietnam and the Western Pacific Region. In addition, the references of selected publications were reviewed, and co-authors suggested additional relevant papers. The term ‘acute respiratory tract infection (ARTI)’ describes all acute infections that involve the lungs or airways (upper and lower). The World Health Organization (WHO) defines ‘pneumonia’ as a child with tachypnoea, with or without signs of respiratory distress. Given the huge overlap in how these terms are applied in clinical practice, the authors considered ‘pneumonia’ and acute lower respiratory tract infection (LRTI) to be synonymous for the purpose of this review.

## Common bacterial pathogens

Bacteria classically described to cause community-acquired pneumonia in children include *Streptococcus pneumoniae, Haemophilus influenzae* type b (Hib) and *Staphylococcus aureus* [6]. Table 1 presents an overview of pathogens commonly associated with acute LRTIs in children less than 5 years of age. With enhanced diagnostic tools, respiratory viruses and atypical bacteria such as *Mycoplasma pneumonia* are commonly detected in children with community-acquired pneumonia, particularly in studies from the Western Pacific Region [7–9]. This review focused on bacterial pathogens because they are the major cause of pneumonia-related mortality and the primary indication for antibiotic use. However, it is important to appreciate that accurate differentiation between viral and/or bacterial infection is a major challenge to clinicians and complicates management [10]. This review provides a brief overview of the most common bacterial pathogens and their reported drug resistance profiles from surveys conducted in the Western Pacific Region.

**Table 1:** Pathogens commonly associated with pneumonia or acute lower respiratory tract infection in children less than 5 years of age^e^

Age group	Bacteria	Viruses
<5 years	*Streptococcus pneumoniae* ^a^ *Haemophilus influenzae* ^a^ *Mycoplasma pneumoniae* ^b^ *Staphylococcus aureus* *Klebsiella pneumoniae* *Streptococcus pyogenes* *Bordetella pertussis* ^c^ *Mycobacterium tuberculosis* ^d^	RSV Rhinovirus Influenza virus (A and B) Parainfluenza virus Human metapneumovirus Adenovirus Human corona virus
Neonates	Group B streptococcus *Listeria monocytogenes* Enteric (gram negative) bacteria *Chlamydia trachomatis*	

*RSV* Respiratory Syncytial Virus, *Staphylococcus aureus* includes methicillin resistant strains (MRSA); *Haemophilus influenzae* includes type b and other encapsulated strains

^a^Disease greatly reduced in settings with universal access to conjugated vaccines; ^b^Typically considered as “atypical bacteria” requiring macrolide therapy; ^c^mainly in unvaccinated babies, in older children it can present as a chronic cough; ^d^The risk of tuberculosis is dependent on the likelihood of *Mycobacteria tuberculosis* exposure/infection, which is a particular problem in areas with uncontrolled tuberculosis transmission

^e^Adapted from [3, 6]

### Streptococcus pneumoniae

The development of resistance mutations against multiple antibiotics is well documented in *S. pneumoniae*, as well as the expansion of resistant clones under antibiotic pressure. Resistance has been recorded against penicillin, tetracycline, cotrimoxazole, chloramphenicol, fluoroquinolones and macrolides [5, 11, 12]. Although the correlation between *in vitro* and *in vivo* resistance remains contentious, it is generally assumed that penicillin (at adequate dosages) should still be effective against pneumococcal pneumonia (not meningitis) caused by strains with low or intermediate levels of resistance *in vitro* [13]. This is difficult to verify, as many factors that influence the outcome of pneumonia treatment—such as underlying comorbid conditions, disease severity and supportive treatment—are poorly described in historical datasets and adequately powered head-to-head comparisons are rare [14–16]. Another important factor to take into consideration is the roll-out of pneumococcal conjugate vaccines. Routine administration of pneumococcal conjugate vaccine (PCV) in infancy has led to major reductions in pneumonia hospitalization and invasive pneumococcal disease in young children [17]. Given that the most common drug resistant strains were included in 7- and 13-valent pneumococcal conjugate vaccines, drug resistant pneumococcal disease has been greatly reduced in areas with high vaccine uptake [18], while reductions in strain carriage also reduced secondary pneumonia cases among older adults [17]. However, recent studies from Canada and the United Kingdom demonstrated substantial increases in multi-drug resistant non-vaccine serotypes in both colonizing and invasive strains since introducing PCV13, with similar findings for colonizing strains after introducing PCV7 in Korean children [19–21].

High penicillin and macrolide resistance rates have been reported in published surveys, but many of these surveys included select patient populations and used minimal inhibitory concentration (MIC) breakpoints with variable stringency, and were also done prior to routine PCV delivery [5, 22, 23]. The use of variable MIC breakpoints in different studies is a major source of confusion and complicates study comparison. MIC breakpoints defined by the Clinical and Laboratory Standards Institute (CLSI) are widely used in the United States, while Europe has adopted the European Committee on Antimicrobial Susceptibility Testing (EUCAST) standards. They use broadly similar *in vitro* methods and specify MIC breakpoints by considering the pharmacokinetic–pharmacodynamic (PK-PD) properties of the drug, the clinical site of disease and the specific mechanism of drug resistance [24]. Studies have shown reasonable comparability between antibiotic susceptibility profiles using revised CLSI and EUCAST breakpoints [25, 26], but some significant differences remain. Against *S. pneumoniae* and *H. influenzae*, cefuroxime and cefaclor breakpoints still produce divergent results [26]. Global surveillance efforts would be greatly enhanced if uniform surveillance criteria can be agreed upon and MIC breakpoint definitions harmonized.

A study conducted in 11 Asian countries reported a low (0.7%; 365/2184) prevalence of penicillin resistance in *S. pneumoniae* from non-meningeal isolates, [12] but it used an MIC breakpoint of ≥8 μg/ml as recommended by CLSI, which is much higher than the >0.06 μg/ml breakpoint proposed by EUCAST. A small case series of children with S. *pneumoniae* septicemia in China reported high drug resistance rates (96%; 24/25) [23], but MIC breakpoints were not specified. In Malaysia, 18% (5/28) of *S. pneumoniae* isolated from blood in children with community-acquired pneumonia was resistant to penicillin (using revised CLSI breakpoints) [27]. Since nasopharyngeal carriage may create a reservoir of resistant *S. pneumoniae* clones, it is important to monitor resistance in carriage specimens as well [28]. Nasopharyngeal *S. pneumoniae* carriage has been reported in 17% (102/614) of healthy Chinese children; 51% were resistant to macrolides by the E-test method [29]. In rural Vietnam, *S. pneumoniae* carriage has been detected in 50% of children, with higher rates in those less than 2 years of age (6–23 months). The resistance rate to amoxicillin and benzylpenicillin was low (4%; 17/421), based on revised CLSI breakpoints, but 95% were resistant to at least one antibiotic (cotrimoxazole 78%, erythromycin 70%, ciprofloxacin 28%) [30]. A more recent Vietnamese survey confirmed excellent penicillin susceptibility in *S. pneumoniae* isolates from respiratory specimens (penicillin 87% using revised CLSI breakpoints), although cephalosporin and macrolide susceptibility was poor (cefuroxime 19%, cefaclor 8%; azithromycin 4%) [31]. This supports the Vietnam national guidance to use high dose (90 mg/kg/day) amoxicillin as first-line treatment of community-acquired pneumonia in children [13].

### Haemophilus influenzae

Although the prevalence of *H. influenzae* type b (Hib) has declined dramatically with widespread roll-out of conjugated Hib vaccine [32, 33], it remains prevalent in settings with poor vaccine uptake. B-lactamase production is commonly associated with Hib infection. In Vietnam, 41% of respiratory Hib isolates produced β-lactamase and 14% were β-lactamase non-producing ampicillin-resistant [31]. However, it is impossible to differentiate clinical infection from asymptomatic colonization using respiratory specimens from non-sterile sites. In China, Hib carriage decreased from 36% in 2000 to 19% in 2012, but β-lactamase-producing isolates increased from 4 to 31% over the same time period. Amoxicillin/clavulanic acid and 2nd or 3rd generation cephalosporins remained universally effective [34]. Despite the national Hib vaccine roll-out in Vietnam, Hib remains a common invasive pathogen [31], but this is expected to decrease as vaccine uptake improves. With increased vaccination uptake, the role of other encapsulated *H. influenzae* strains have increased [35]. A study in China found that 100% of *H. influenzae* strains identified in the sputum of children with pneumonia were non-typable; 1% (2/279) were β-lactamase-positive and 5% were β-lactamase non-producing ampicillin-resistant or intermediately resistant [36].

### Staphylococcus aureus

*S. aureus* remains a common cause of community-acquired pneumonia. Pioneering lung puncture studies performed in Chile and Papua New Guinea identified *S. aureus* as a common pathogen in children with community-acquired pneumonia [37]. More recent studies found the pathogen predominantly in children at the severe end of the pneumonia disease spectrum, with increased frequency in severely malnourished children [38, 39]. *S. aureus* also poses particular problems following influenza or measles infection, and as a secondary infection in hospitalized children [38, 40, 41]. A major challenge has been the emergence of methicillin-resistant *S. aureus* (MRSA) and more recently, a decrease in vancomycin susceptibility [42, 43]. Among positive *S. aureus* blood cultures in Australian and New Zealand children, MRSA has been reported in 13% (142/1,073) with three times the average rate (incidence rate ratio, 3 [95%, CI: 2–4]), found among Aboriginal and Pacific Islander populations [44, 45]. A recent Malaysian survey reported MRSA in 8% (3/38) of children with community-acquired S. *aureus* bacteremia; 32% had skin or soft tissues infections and 32% had community-acquired pneumonia [27]. A study assessing the prevalence of heterogeneous vancomycin-intermediately-resistant *S. aureus* (hVISA*)* in Asian countries (including South Korea, Taiwan, Hong Kong, Thailand, the Philippines, Vietnam, India and Sri Lanka) reported that among 462 MRSA isolates, 3.5% were hVISA, with the highest prevalence in South Korea and Vietnam (7.0%) [46]. A more extensive report on antibiotic resistance in 15 hospitals throughout Vietnam found MRSA in 20% of children and adults with *S. aureus* bacteremia; vancomycin resistance rates were very low [47].

### Atypical bacteria

*Mycoplasma pneumoniae* is highly prevalent in children diagnosed with pneumonia in the Western Pacific Region [4]. *M. pneumoniae* is inherently resistant to all β-lactams antibiotics and vancomycin, because it does not have a cell wall. With excessive macrolide use in recent years, reported macrolide resistance is near universal (90–100%) in parts of Asia [48]. Most countries report high rates of macrolide resistance (Japan 89% [49, 50], China 83–98% [5, 51, 52], South Korea 63% [53], Hong Kong 47% [54], Taiwan 23% [55]), although the methods for *M. pneumoniae* susceptibility testing are poorly standardized. *M. pneumoniae* remains susceptible to macrolides in Australia where it is less frequently used [56]. Resistance to tetracyclines and fluoroquinolones have not been reported in clinical isolates and may provide a treatment alternative in some children, although reduced *in vitro* susceptibility has been reported [48]. However, most *M. pneumoniae* cases recover either without antibiotic treatment or despite documented drug resistance, thus the clinical value of antibiotic treatment remains uncertain [57].

Treatment of ARTIs is a major driver of antibiotic use in children. It is important for clinicians to be familiar with the most common bacterial causes, their local drug resistance profiles and the likely impact of antibiotic therapy (both positive and negative) in order to develop a rational treatment approach. Because a timely and definitive bacteriological diagnosis is currently impossible, empiric antibiotic treatment is justified in any acutely ill child. However, there is a need to critically consider factors that promote unnecessary and irrational antibiotic use, especially in children who are not acutely ill.

## Factors promoting irrational antibiotic use

Antibiotic use generates selective pressure that increases the prevalence of drug resistant strains; hence, strategies to improve rational antibiotic use are important to protect antibiotics as a precious resource. A study of antibiotic use in Vietnamese hospitals showed that a large proportion of in-patients received inappropriate antibiotic therapy [58]. The main factors that promote unnecessary antibiotic use in the Western Pacific Region, using Vietnam as an exemplar, are listed below.

### Unrestricted antibiotic access

Given unrestricted access to over-the-counter antibiotics, treatment of any respiratory infection with antibiotics is a common practice in Vietnam, Malaysia, and South Korea [59]. In Vietnam, the *Pharmaceutical Law* passed in 2005 requires an antibiotic prescription, but 38% of caregivers still access antibiotics without any formal medical assessment [60]; even injectable antibiotics can be acquired at local shops without prescription [61]. In the Western Pacific Region, antibiotic use before presentation to a doctor is common; more than 40% in Mongolia [62] and more than 50% of children admitted with ARTIs in the Philippines [63]. A study in nine international sites—Colombia, Ghana, India, Mexico, Pakistan, South Africa (2 sites), Vietnam and Zambia—found that the use of antibiotics in the 48 h prior to hospital admission was associated with treatment failure (OR: 1.8; 95% CI: 1.27–2.66) [64]. In addition, agricultural use of antibiotics remains essentially unregulated in most Asian countries, with high rates of colistin and cephalosporin use in the pig and poultry industries, resulting in increased rates of drug-resistant infections in human populations [65, 66].

### Unrealistic expectations and limited awareness

Limited awareness among the general public (including politicians) is a challenge in all settings; the WHO recently launched a program to increase awareness about antimicrobial resistance and the need for more prudent antibiotic use [59]. In response, country-specific strategies to limit antimicrobial resistance have been launched in the United Kingdom, the United States and Australia [67–69], but few Asian countries have followed suit. Unrealistic public expectation is a major factor driving excessive antibiotic use [59, 70]. A study in rural Vietnam showed that just 13% of caregivers had correct knowledge about acute respiratory infections and 38% of caregivers self-managed common colds by buying antibiotics without prescription at the local pharmacy [60]. In Malaysia, 67% of people believed antibiotics to be effective against viral infections, with 47% using antibiotics during a common cold [61]. Moreover, antibiotic use is strongly influenced by cultural preferences and beliefs. Patients in Vietnam and China believe injectable antibiotics are more potent than oral options, and readily access injectable antibiotics without prescription [71]. A lack of adequate knowledge is also a problem among healthcare providers. A study in Vietnam demonstrated poor awareness about the risks and consequences of drug resistance among rural health-care providers when treating ARTIs; only 19% complied with recommended guidelines and 79% used antibiotics for common colds [72].

### Physician-related factors

Table 2 provides an overview of physician-related factors that explains some of the excessive antibiotic use seen in Vietnam and parts of the Western Pacific Region. The difficulty in accurately differentiating viral from bacterial pneumonia is a major challenge in all settings. A study in Finland showed that of the routine blood tests, only the C-reactive protein (CRP) level differed significantly between bacterial and viral pneumonia patients [10]. Most children with dense lobar infiltrates on chest radiograph had laboratory evidence of a bacterial infection, but interstitial infiltrates were seen in both viral and bacterial pneumonia [10]. A recent randomized-controlled trial in Vietnam showed that point-of-care CRP testing reduced inappropriate antibiotic use for non-severe ARTIs [73]. A previous randomized-controlled trial in China investigated whether serum pro-calcitonin (PCT) could reduce unnecessary antibiotic use [74], but although the mean duration of antibiotic treatment was shorter in the PCT group, the antibiotic prescription rate was higher. It is hoped that rapid point-of-care testing for common respiratory viruses and biomarkers of severe bacterial infection will offer better bedside guidance in the near future, assisting more appropriate antibiotic use.

**Table 2:** Physician related factors that contribute to excessive antibiotic use in the Western Pacific Region

Factor identified	Examples from the Western Pacific Region
Professional hierarchy	• Junior physicians adopt the prescription habits of senior physicians without rigorous discussion or review of the evidence [86]. • In Vietnam, inappropriate antibiotic use is a particular problem in obstetrics, gynecology and surgery wards where professional hierarchy is most pronounced [58]
No consideration of “societal risk”	• Doctors and patients often prefer newer and more expensive antibiotics, which are considered more “powerful” [62] • Physicians provide antibiotics to help individual patients; potential societal risks are not considered [86]; • In the absence of functional microbiology services, physicians have limited information on local drug-resistance profiles and the impact of excessive antibiotic use;
Perceived patient/parent expectation	• Doctors strive for patient satisfaction and if patients request antibiotics it is usually prescribed [70, 87]; In Korea, 73% of doctors prescribe antibiotics for a common cold if requested by parents [88]; In Malaysia, 67% of patients believe that antibiotics help for viral infections [61] • Doctors have no time or motivation to explain the rationale for not using antibiotics
Fear of poor patient outcome or litigation	• Fear of poor patient outcomes is often listed as a key motivation for the use of broad-spectrum antibiotics by doctors [58, 59, 89] • Fear of litigation is not yet a major driver in the Western Pacific, but is likely to become a more prominent factor with increased development [90]
Inadequate microbiology services	• Near universal use of empiric broad spectrum antibiotics is common in places with poor microbiology services [70, 87]. • In Vietnam, antibiotic use was reduced in hospitals with functional microbiology laboratories [58]
Financial incentives to use antibiotics	• Doctors’ prescribing habits is influenced by personal income generated and incentives provided by pharmaceutical companies [70, 86, 87]. In China, as in many other Western Pacific countries, drug prescriptions supplement a doctor’s income [62]. • In South-Korea drug dispensing by health care workers was banned in 2000, resulting in major reductions in antibiotic use [62]

Because most child pneumonia deaths are caused by bacterial pathogens, current WHO guidelines recommend antibiotic use in all pneumonia cases, as defined by the presence of fast breathing. This approach may encourage the overuse of antibiotics, especially in children without danger signs who present with wheezing as a sign of reactive airway disease, which usually indicates a viral infection. Audible wheezing has been noted in 49% of Vietnamese children admitted with ‘pneumonia’ [75], which may identify a subgroup that does not require antibiotics [76]. WHO guidelines previously advised intravenous antibiotics in all children diagnosed with severe pneumonia (signs of respiratory distress), but oral amoxicillin was found to be effective in 92% (948/1,025) of children with a clinical diagnosis of severe pneumonia in Pakistan [77]. Subsequent trials demonstrated that home-based treatment can be applied to a wide variety of settings [74]. A multi-center study conducted in Bangladesh, Egypt, Ghana and Vietnam reported 9% (95% CI: 7–11%) treatment failure with 5 days of high dose oral amoxicillin (80–90 mg/kg/day), varying from 6% (95% CI: 3–10%) in Ghana to 13% (95% CI: 8–18%) in Vietnam [75]. A sizeable (but unknown) proportion of cases enrolled in these “clinical pneumonia” studies would have had viral infections. Therefore, it is not clear how much “treatment failure” actually resulted from inadequate antibiotic treatment for bacterial pneumonia; most children found to be “unresponsive to antibiotics” probably had viral pneumonia [78, 79]. Despite the available evidence, most doctors in Vietnam routinely hospitalize children with “clinical pneumonia” to administer intravenous antibiotics; unnecessary hospitalization increases both healthcare cost and the risk of nosocomial infection.

## Proposed recommendations

Actions and recommendations to improve rational antibiotic use in both the community and hospital environment are summarized in Table 3. Unrestricted antibiotic access and self-medication are firmly entrenched in Vietnam and most other Asian countries [80]. Given the multiple vested interests that protect the status quo, strong regulation and effective law enforcement will be required to limit excessive antibiotic use. In addition to public education programs, better training of healthcare providers to critically review the need for antibiotic use is essential [59]. Only 31% of countries in the Western Pacific Region report high awareness of antimicrobial resistance among their healthcare providers [59]. An educational program in Indonesia, including face-to-face clinician visits and group discussions, significantly reduced the use of injectable drugs [81], but has not been replicated elsewhere.

**Table 3:** Actions and recommendations to improve rational antibiotic use

Actions	Suggested high-level recommendations^a^
In general	
Enhanced regulation	• Limit over-the-counter availability of antibiotics; establish strong national policies for appropriate antibiotic regulation; implement measures to ensure compliance • Improved national surveillance of antimicrobial resistance and adherence to treatment guidelines • Regulate agricultural use of antibiotics
Patient/public education	• Increase general awareness of adverse effects associated with excessive antibiotic use • Educate parents, caretakers, politicians and the general community about the benefits of restricted antibiotic use
Universal Hib and PCV	• Make Hib and PCV universally available free of charge • Maintain high uptake of other vaccines (e.g. pertussis and measles)
Within hospitals	
Provide information on local drug resistance patterns	• Maintain a network of functional microbiology laboratories, with adequate quality assurance and sharing of information
Establish functional antimicrobial stewardship programs	• Each hospital should have an antimicrobial stewardship program and a Drug and Therapeutics Committee with access to reliable and up-to-date data on antibiotic usage and drug resistance profiles • Each hospital should have regular/annual antibiotic use audits led by pharmacists or infectious disease control personnel
Provide clear guidance	• Develop national/regional consensus treatment guidelines that consider the international evidence base, as well as local disease etiology and drug resistance data
Eliminate perverse incentives	• Delink remuneration from antibiotic prescription • Ban incentives to doctors to provide antibiotics or use specific medical products
Educate medical students and trainees	• Include antimicrobial stewardship in the undergraduate medical, nursing and pharmacy curriculum • Highlight the growing drug resistance problem and need for prudent use

*PCV* pneumococcal conjugate vaccine, *Hib Haemophilus influenzae* type b

^a^In addition, a detailed assessment of pneumonia case management should be conducted to understand clinical decision-making and provide more pragmatic guidance to clinicians in the field. Exemplars of comprehensive national strategies to address antimicrobial resistance and limit excessive antibiotic use include the United Kingdom, the United States and Australia [67–69]

In 2013, the WHO facilitated a meeting of Western Pacific Region countries in Manila to identify feasible antimicrobial resistance (AMR) strategies [59], but this has not yet translated into revised childhood pneumonia guidance or management practices. In Vietnam, the Vietnam Resistance (VINARES) project aims to strengthen laboratory surveillance and reduce irrational antibiotic use. The project covers 16 participating hospitals [82], but outside of the participating hospitals AMR surveillance remains weak. Laboratory capacity to assist microbiological diagnosis and guide clinical management is insufficient throughout the region [83]. Functional antibiotic stewardship programs have been established in some countries, but this should become standard practice and be adapted to the local context [44]. An antibiotic stewardship program in Chinese hospitals set targets for antibiotic prescriptions and penalized doctors who prescribed antibiotics inappropriately [84]. After 2 years, antibiotic prescriptions decreased by 58–68% in inpatients and 15–25% in outpatients [85]. An antimicrobial stewardship program is currently being implemented in Vietnam (through VINARES), but the scope of the program is limited and there is a need for similar programs, including regular audits of antibiotic use, in every hospital [82].

## Conclusion

Optimal child pneumonia management presents an opportunity to reduce excessive antibiotic use in the Western Pacific Region. However, encouraging the rational use of antibiotics requires education of healthcare professionals, facilitation of cultural change, improved clinical guidance and the establishment of functional microbiology laboratories to monitor disease etiology and drug resistance patterns, together with the removal of inappropriate incentives and effective enforcement of national regulations to restrict antibiotic use in healthcare and agriculture.

## Abbreviations

ARTI: Acute respiratory tract infection
AMR: Antimicrobial resistance
CRP: C-reactive protein
CLSI: Clinical and Laboratory Standards Institute
CI: Confidence interval
EUCAST: European Committee on Antimicrobial Susceptibility Testing
*H. influenzae*: *Haemophilus influenzae*
Hib: *Haemophilus influenzae* type b
hVISA: Heterogeneous vancomycin-intermediate S.*aureus*
MRSA: Methicillin resistant *staphylococcus aureus*
MIC: Minimal inhibitory concentration
M. *pneumoniae*: *Mycoplasma pneumonia*
PK-PD: Pharmacokinetic-pharmacodynamic
PCV: Pneumococcal conjugate vaccine
PCT: Procalcitonin
S. *aureus*: *Staphylococcus aureus*
S. *pneumoniae*: *Streptococcus pneumoniae*
VINARES: Vietnam resistance project
WHO: World Health Organization

## References

[1] Walker CLF, Rudan I, Liu L, Nair H, Theodoratou E, Bhutta ZA, O’Brien KL, Campbell H, Black RE (2013). Global burden of childhood pneumonia and diarrhoea. Lancet.

[2] WHO. World Health Statistics 2016: Monitoring health for the SDGs. 2016. http://www.who.int/gho/publications/world_health_statistics/2016/en/. Accessed 1 Feb 2017.

[3] Naor B-Z, Carolyn M, John M, Emmalita M, Marianna T. Integrated Management of Childhood Illness (IMCI) implementation in the Western Pacific Region: information package. World Health Organ WPG. 2013. http://www.wpro.who.int/child_adolescent_health/documents/imci_info_package/en/. Accessed 5 Feb 2017.

[4] Nguyen TKP, Tran TH, Robert CL, Graham SM, Marais BJ. Child pneumonia in the Western Pacific Region. Paediatr Respir Rev. 2017;21:102–10. 10.1016/j.prrv.2016.07.004 PMC7106312 27569107

[5] Felmingham D, Feldman C, Hryniewicz W, Klugman K, Kohno S, Low D, Mendes C, Rodloff A (2002). Surveillance of resistance in bacteria causing community‐acquired respiratory tract infections. Clin Microbiol Infect.

[6] McIntosh K (2002). Community-acquired pneumonia in children. N Engl J Med.

[7] Huong PLT, Hien PT, Lan NTP, Binh TQ, Tuan DM, Anh DD (2014). First report on prevalence and risk factors of severe atypical pneumonia in Vietnamese children aged 1–15 years. BMC Public Health.

[8] Wu Z, Li Y, Gu J, Zheng H, Tong Y, Wu Q (2014). Detection of viruses and atypical bacteria associated with acute respiratory infection of children in Hubei. China Respirology.

[9] Yoshida L-M, Suzuki M, Thiem VD, Smith WP, Tsuzuki A, Huong VTT, Takahashi K, Miyakawa M, Anh NTH, Watanabe K (2014). Population based cohort study for pediatric infectious diseases research in Vietnam. Trop Med health.

[10] Virkki R, Juven T, Rikalainen H, Svedström E, Mertsola J, Ruuskanen O (2002). Differentiation of bacterial and viral pneumonia in children. Thorax.

[11] Fuller JD, Low DE (2005). A review of Streptococcus pneumoniae infection treatment failures associated with fluoroquinolone resistance. Clin Infect Dis.

[12] Kim SH, Song J-H, Chung DR, Thamlikitkul V, Yang Y, Wang H, Lu M, So TM-K, Hsueh P-R, Yasin RM (2012). Changing trend of antimicrobial resistance and serotypes in Streptococcus pneumoniae in Asian countries: an ANSORP study. Antimicrob Agents Chemother.

[13] Peterson LR (2006). Penicillins for treatment of pneumococcal pneumonia: does in vitro resistance really matter?. Clin Infect Dis.

[14] Metlay JP (2002). Update on community-acquired pneumonia: impact of antibiotic resistance on clinical outcomes. Curr Opin Infect Dis.

[15] Feldman C, Anderson R (2006). Controversies in the treatment of pneumococcal community-acquired pneumonia. Future Microbiol.

[16] Metlay JP, Singer DE (2002). Outcomes in lower respiratory tract infections and the impact of antimicrobial drug resistance. Clin Microbiol Infect.

[17] Oliwa JN, Marais BJ. Vaccines to prevent pneumonia in children–a developing country perspective (Epub ahead of print). Paediatr Respir Rev. 2017;22:23–30. 10.1016/j.prrv.2015.08.004 PMC6995362 26364006

[18] Dagan R (2003). Antibiotic resistance and the potential impact of pneumococcal conjugate vaccines. Commun Dis Intell Q Rep.

[19] Duvvuri VR, Deng X, Teatero S, Memari N, Athey T, Fittipaldi N, Gubbay JB (2016). Population structure and drug resistance patterns of emerging non-PCV-13 Streptococcus pneumoniae serotypes 22 F, 15A, and 8 isolated from adults in Ontario, Canada. Infect Genet Evol.

[20] Sheppard C, Fry N, Mushtaq S, Woodford N, Reynolds R, Janes R, Pike R, Hill R, Kimuli M, Staves P (2016). Rise of multidrug-resistant non-vaccine serotype 15A Streptococcus pneumoniae in the United Kingdom, 2001 to 2014. Euro Surveill.

[21] Lee S, Kim J-H, Kim S-H, Park M, Bae S (2013). Prevalent multidrug-resistant nonvaccine serotypes in pneumococcal carriage of healthy Korean children associated with the low coverage of the seven-valent pneumococcal conjugate vaccine. Osong Public Health Res Perspect.

[22] Low DE, Pichichero ME, Schaad UB (2004). Optimizing antibacterial therapy for community-acquired respiratory tract infections in children in an era of bacterial resistance. Clin Pediatr.

[23] Su X-Y, Wen S-H, Lin L, Li C-C (2013). Clinical characteristics of children with Streptococcus pneumoniae septicemia and drug sensitivity of Streptococcus pneumoniae. Zhongguo dang dai er ke za zhi (Chinese journal of contemporary pediatrics).

[24] Marchese A, Esposito S, Barbieri R, Bassetti M, Debbia E (2012). Does the adoption of EUCAST susceptibility breakpoints affect the selection of antimicrobials to treat acute community-acquired respiratory tract infections?. BMC Infect Dis.

[25] Kahlmeter G (2015). The 2014 Garrod Lecture: EUCAST–are we heading towards international agreement?. J Antimicrob Chemother.

[26] Kassim A, Omuse G, Premji Z, Revathi G (2016). Comparison of Clinical Laboratory Standards Institute and European Committee on Antimicrobial Susceptibility Testing guidelines for the interpretation of antibiotic susceptibility at a University teaching hospital in Nairobi, Kenya: a cross-sectional study. Ann Clin Microbiol Antimicrob.

[27] Kaur AA (2016). Community-acquired bacteremia in Paediatrics: Epidemiology, aetiology and patterns of antimicrobial resistance in a Tertiary Care Centre, Malaysia. Med J Malaysia.

[28] Samore MH, Magill MK, Alder SC, Severina E, Morrison-de Boer L, Lyon JL, Carroll K, Leary J, Stone MB, Bradford D (2001). High rates of multiple antibiotic resistance instreptococcus pneumoniae from healthy children living in isolated rural communities: association with cephalosporin use and intrafamilial transmission. Pediatrics.

[29] Hu J, Sun X, Huang Z, Wagner AL, Carlson B, Yang J, Tang S, Li Y, Boulton ML, Yuan Z (2016). Streptococcus pneumoniae and Haemophilus influenzae type b carriage in Chinese children aged 12–18 months in Shanghai, China: a cross-sectional study. BMC Infect Dis.

[30] Hoa NQ, Trung NV, Larsson M, Eriksson B, Phuc HD, Chuc NT, Lundborg CS (2010). Decreased Streptococcus pneumoniae susceptibility to oral antibiotics among children in rural Vietnam: a community study. BMC Infect Dis.

[31] Van P, Binh P, Minh N, Morrissey I, Torumkuney D (2016). Results from the Survey of Antibiotic Resistance (SOAR) 2009–11 in Vietnam. J Antimicrob Chemother.

[32] Chen WJ, Moulton LH, Saha SK, Mahmud AA, Arifeen SE, Baqui AH (2014). Estimation of the herd protection of Haemophilus influenzae type b conjugate vaccine against radiologically confirmed pneumonia in children under 2 years old in Dhaka, Bangladesh. Vaccine.

[33] Flasche S, Takahashi K, Vu DT, Suzuki M, Nguyen TH-A, Le H, Hashizume M, Dang DA, Edmond K, Ariyoshi K (2014). Early indication for a reduced burden of radiologically confirmed pneumonia in children following the introduction of routine vaccination against Haemophilus influenzae type b in Nha Trang, Vietnam. Vaccine.

[34] Zhu H, Wang A, Tong J, Yuan L, Gao W, Shi W, Yu S, Yao K, Yang Y (2015). Nasopharyngeal carriage and antimicrobial susceptibility of Haemophilus influenzae among children younger than 5 years of age in Beijing, China. BMC Microbiol.

[35] Van Eldere J, Slack MP, Ladhani S, Cripps AW (2014). Non-typeable Haemophilus influenzae, an under-recognised pathogen. Lancet Infect Dis.

[36] Hu J, Wang X, Ai T, Xie X, Liu X, Liu H, Yang L, Li H, Yang T, Zhang T (2016). Multicenter prospective epidemiological studies on Haemophilus influenzae infection among hospitalized children with lower respiratory tract infections. Zhonghua er ke za zhi (Chinese journal of pediatrics).

[37] Mimica I, Donoso E, Howard JE, Ledermann GW (1971). Lung puncture in the etiological diagnosis of pneumonia: a study of 543 infants and children. Amer J Dis Child.

[38] Asghar R, Banajeh S, Egas J, Hibberd P, Iqbal I, Katep-Bwalya M, Kundi Z, Law P, MacLeod W, Maulen-Radovan I (2008). Multicentre randomized controlled trial of chloramphenicol vs. ampicillin and gentamicin for the treatment of very severe pneumonia among children aged 2 to 59 months in low resource settings: a multicenter randomized trial (spear study). BMJ.

[39] Chisti MJ, Graham SM, Duke T, Ahmed T, Faruque ASG, Ashraf H, Bardhan PK, Shahid AS, Shahunja K, Salam MA (2014). Post-discharge mortality in children with severe malnutrition and pneumonia in bangladesh. PLoS One.

[40] Hughes AJ, Ariffin N, Huat TL, Molok HA, Hashim S, Sarijo J, Latif NHA, Hanifah YA, Kamarulzaman A (2005). Prevalence of nosocomial infection and antibiotic use at a university medical center in Malaysia. Infect Control Hosp Epidemiol.

[41] Lee M, Chiu C, Chow V, Lam R, Lai R (2007). Prevalence of hospital infection and antibiotic use at a university medical center in Hong Kong. J Hosp Infect.

[42] Howden BP, Davies JK, Johnson PD, Stinear TP, Grayson ML (2010). Reduced vancomycin susceptibility in Staphylococcus aureus, including vancomycin-intermediate and heterogeneous vancomycin-intermediate strains: resistance mechanisms, laboratory detection, and clinical implications. Clin Microbiol Rev.

[43] Song J-H, Hsueh P-R, Chung DR, Ko KS, Kang C-I, Peck KR, Yeom J-S, Kim S-W, Chang H-H, Kim Y-S (2011). Spread of methicillin-resistant Staphylococcus aureus between the community and the hospitals in Asian countries: an ANSORP study. J Antimicrob Chemother.

[44] NPS Medicinewise. Antimicrobial resistance - it's happening right now. 2014. http://www.nps.org.au/publications/health-professional/health-news-evidence/2014/antimicrobial-resistance. Accessed 5 Feb 2017.

[45] McMullan BJ, Bowen A, Blyth CC, Van Hal S, Korman TM, Buttery J, Voss L, Roberts S, Cooper C, Tong SY. Epidemiology and Mortality of Staphylococcus aureus Bacteremia in Australian and New Zealand Children. JAMA Pediatr. 2016;170(10):979–86. 10.1001/jamapediatrics.2016.1477 27533601

[46] Chung DR, Lee C, Kang YR, Baek JY, Kim SH, Ha YE, Kang C-I, Peck KR, Lee NY, Song J-H (2015). Genotype-specific prevalence of heterogeneous vancomycin-intermediate Staphylococcus aureus in Asian countries. Int J Antimicrob Agents.

[47] Vietnam Ministry of Health. First report on antibiotics use and resistance in Vietnam. 2009. http://benhnhietdoi.vn/su-dung-khang-sinh/. Accessed 3 Feb 2017.

[48] Pereyre S, Goret J, Bébéar C (2016). Mycoplasma pneumoniae: current knowledge on macrolide resistance and treatment. Front Microbiol.

[49] Bell DM (2001). Promoting appropriate antimicrobial drug use: perspective from the Centers for Disease Control and Prevention. Clin Infect Dis.

[50] Matsuda K, Narita M, Sera N, Maeda E, Yoshitomi H, Ohya H, Araki Y, Kakuma T, Fukuoh A, Matsumoto K (2013). Gene and cytokine profile analysis of macrolide-resistant Mycoplasma pneumoniae infection in Fukuoka, Japan. BMC Infect Dis.

[51] Bao F, Qu J, Liu Z, Qin X, Cao B (2013). The clinical characteristics, treatment and outcome of macrolide-resistant Mycoplasma pneumoniae pneumonia in children. Zhonghua jie he he hu xi za zhi (Chinese journal of tuberculosis and respiratory diseases).

[52] Zhou Z, Li X, Chen X, Luo F, Pan C, Zheng X, Tan F (2015). Macrolide-resistant Mycoplasma pneumoniae in adults in Zhejiang, China. Antimicrob Agents Chemother.

[53] Hong KB, Choi EH, Lee HJ, Lee SY, Cho EY, Choi JH, Kang HM, Lee J, Ahn YM, Kang Y-H (2013). Macrolide resistance of Mycoplasma pneumoniae, South Korea, 2000–2011. Emerg Infect Dis.

[54] Ho P-L, Law PY, Chan BW, Wong C-W, To KK, Chiu SS, Cheng VC, Yam W-C (2015). Emergence of macrolide-resistant Mycoplasma pneumoniae in Hong Kong is linked to increasing macrolide resistance in multilocus variable-number tandem-repeat analysis type 4-5-7-2. J Clin Microbiol.

[55] Wu PS, Chang LY, Lin HC, Chi H, Hsieh YC, Huang YC, Liu CC, Huang YC, Huang LM (2013). Epidemiology and clinical manifestations of children with macrolide‐resistant Mycoplasma pneumoniae pneumonia in Taiwan. Pediatr Pulmonol.

[56] Xue G, Wang Q, Yan C, Jeoffreys N, Wang L, Li S, Gilbert GL, Sun H (2014). Molecular characterizations of PCR-positive Mycoplasma pneumoniae specimens collected from Australia and China. J Clin Microbiol.

[57] Spuesens EB, Sauteur PMM, Vink C, van Rossum AM (2014). Mycoplasma pneumoniae infections–Does treatment help?. J Infect.

[58] Thu TA, Rahman M, Coffin S, Harun-Or-Rashid M, Sakamoto J, Hung NV (2012). Antibiotic use in Vietnamese hospitals: a multicenter point-prevalence study. Am J Infect Control.

[59] WHO. Antimicrobial resistance: global report on surveillance. 2014. http://www.who.int/drugresistance/documents/surveillancereport/en/. Accessed 19 Jan 2017.

[60] Hoa NQ, Chuc NTK, Phuc HD, Larsson M, Eriksson B, Lundborg CS (2011). Unnecessary antibiotic use for mild acute respiratory infections during 28-day follow-up of 823 children under five in rural Vietnam. Tran R Soc Trop Med Hyg.

[61] Nordberg P, Stalsby-Lundborg C, Tomson G (2005). Consumers and providers-Could they make better use of antibiotics?. Int J Risk Saf Med.

[62] WHO. Antimicrobial Resistance in the Western Pacific region: A review of surveillance and Health system response. 2015. http://www.wpro.who.int/entity/drug_resistance/documents/amr_wpr.pdf. Accessed 9 Feb 2017.

[63] Capeding MR, Bravo L, Santos J, Kilgore PE, Kim SA, Balter I, Hubler R, Ye J, Moscariello M (2013). Prospective surveillance study of invasive pneumococcal disease among urban children in the Philippines. Pediatr Infect Dis.

[64] Addo-Yobo E, Chisaka N, Hassan M, Hibberd P, Lozano JM, Jeena P, MacLeod WB, Maulen I, Patel A, Qazi S (2004). Oral amoxicillin versus injectable penicillin for severe pneumonia in children aged 3 to 59 months: a randomised multicentre equivalency study. Lancet.

[65] Nguyen NT, Nguyen HM, Nguyen CV, Nguyen TV, Nguyen MT, Thai HQ, Ho MH, Thwaites G, Ngo HT, Baker S (2016). The use of colistin and other critical antimicrobials on pig and chicken farms in southern Vietnam and their association with resistance in commensal Escherichia coli. Appl Environ Microbiol.

[66] Liu Y-Y, Wang Y, Walsh TR, Yi L-X, Zhang R, Spencer J, Doi Y, Tian G, Dong B, Huang X (2016). Emergence of plasmid-mediated colistin resistance mechanism MCR-1 in animals and human beings in China: a microbiological and molecular biological study. Lancet Infect Dis.

[67] Davies S, Gibbens N. UK five year antimicrobial resistance strategy 2013 to 2018. Lodon: UK Goverment. 2013. https://www.gov.uk/government/uploads/system/uploads/attachment_data/file/244058/20130902_UK_5_year_AMR_strategy.pdf. Accessed 9 Feb 2017.

[68] CDC. Antibiotic resistance threats in the United States, 2013: Centres for Disease Control and Prevention, US Department of Health and Human Services. 2013. https://www.cdc.gov/drugresistance/threat-report-2013/index.html. Accessed 9 Feb 2017.

[69] The Department of Health. Antimicrobial resistance. Australia Government (AMR). 2016. http://www.health.gov.au/internet/main/publishingnsf/Content/ohp-amr.htm. Accessed 9 Feb 2017.

[70] Hulscher ME, van der Meer JW, Grol RP (2010). Antibiotic use: how to improve it?. Int J Med Microbiol.

[71] Mills A, Brugha R, Hanson K, McPake B (2002). What can be done about the private health sector in low-income countries?. Bull World Health Organ.

[72] Hoa NQ, Larson M, Chuc NTK, Eriksson B, Trung NV, Stålsby CL (2009). Antibiotics and paediatric acute respiratory infections in rural Vietnam: health‐care providers’ knowledge, practical competence and reported practice. Trop Med Int Health.

[73] Do NT, Ta NT, Tran NT, Than HM, Vu BT, Hoang LB, van Doorn HR, Vu DT, Cals JW, Chandna A (2016). Point-of-care C-reactive protein testing to reduce inappropriate use of antibiotics for non-severe acute respiratory infections in Vietnamese primary health care: a randomised controlled trial. Lancet Global Health.

[74] Dai B, Yuan X, Liu J (2015). Value of serum procalcitonin for the guidance of antibiotic therapy in children with lower respiratory tract infection. Zhongguo dang dai er ke za zhi (Chinese journal of contemporary pediatrics).

[75] Addo‐Yobo E, Anh DD, El‐Sayed HF, Fox LM, Fox MP, MacLeod W, Saha S, Tuan TA, Thea DM, Qazi S (2011). Outpatient treatment of children with severe pneumonia with oral amoxicillin in four countries: the MASS study. Trop Med Int Health.

[76] Singh V, Aneja S (2011). Pneumonia–management in the developing world. Paediatr Respir Rev.

[77] Hazir T, Fox LM, Nisar YB, Fox MP, Ashraf YP, MacLeod WB, Ramzan A, Maqbool S, Masood T, Hussain W (2008). Ambulatory short-course high-dose oral amoxicillin for treatment of severe pneumonia in children: a randomised equivalency trial. Lancet.

[78] Hazir T, Nisar YB, Abbasi S, Ashraf YP, Khurshid J, Tariq P, Asghar R, Murtaza A, Masood T, Maqbool S (2011). Comparison of oral amoxicillin with placebo for the treatment of World Health Organization–defined nonsevere pneumonia in children aged 2–59 months: a multicenter, double-blind, randomized, placebo-controlled trial in Pakistan. Clin Infect Dis.

[79] Jehan F, Nisar MI, Kerai S, Brown N, Balouch B, Hyder Z, Ambler G, Ginsburg AS, Zaidi AK (2016). A double blind community-based randomized trial of amoxicillin versus placebo for fast breathing pneumonia in children aged 2–59 months in Karachi, Pakistan (RETAPP). BMC Infect Dis.

[80] Van der Geest S, Hardon A (1990). Self-medication in developing countries. J Soc Adm Pharm.

[81] Hadiyono JEP, Suryawati S, Danu SS, Santoso B (1996). Interactional group discussion: results of a controlled trial using a behavioral intervention to reduce the use of injections in public health facilities. Soc Sci Med.

[82] Wertheim HF, Chandna A, Vu PD, Van Pham C, Nguyen PDT, Lam YM, Van Nguyen CV, Larsson M, Rydell U, Nilsson LE (2013). Providing impetus, tools, and guidance to strengthen national capacity for antimicrobial stewardship in Vietnam. PLoS Med.

[83] Lee Y, Wakabayashi M (2013). Key informant interview on antimicrobial resistance (AMR) in some countries in the western pacific region. Global Health.

[84] Yin X, Song F, Gong Y, Tu X, Wang Y, Cao S, Liu J, Lu Z (2013). A systematic review of antibiotic utilization in China. J Antimicrob Chemother.

[85] Xiao Y, Zhang J, Zheng B, Zhao L, Li S, Li L (2013). Changes in Chinese policies to promote the rational use of antibiotics. PLoS Med.

[86] Hulscher ME, Grol RP, van der Meer JW (2010). Antibiotic prescribing in hospitals: a social and behavioural scientific approach. Lancet Infect Dis.

[87] Laxminarayan R, Duse A, Wattal C, Zaidi AK, Wertheim HF, Sumpradit N, Vlieghe E, Hara GL, Gould IM, Goossens H (2013). Antibiotic resistance - the need for global solutions. Lancet Infect Dis.

[88] Cho H-J, Hong S-J, Park S (2004). Knowledge and beliefs of primary care physicians, pharmacists, and parents on antibiotic use for the pediatric common cold. Soc Sci Med.

[89] Murni IK, Duke T, Kinney S, Daley AJ, Soenarto Y (2015). Reducing hospital-acquired infections and improving the rational use of antibiotics in a developing country: an effectiveness study. Arch Dis Child.

[90] Fox C (2011). Resisting antibiotic resistance: legal strategies to maintain man's dominion over microbes. Hous J Health L & Pol'y.

